# 150 years since “The etiology, concept, and prophylasix of childbed fever”

**DOI:** 10.1186/1753-6561-5-S6-O61

**Published:** 2011-06-29

**Authors:** AJ Stewardson, D Pittet

**Affiliations:** 1 Infection Control Program, University of Geneva Hospitals, Geneva, Switzerland

## 

2011 marks the sesquicentennial anniversary of the publication of “The etiology, concept, and prophylaxis of childbed fever”, the *magnum opus* of Ignaz Philipp Semmelweis' (1818–1865) determined, but not infrequently self-defeating, efforts to prove his theories regarding this disease. Now widely regarded as “the father of infection control”, his struggle to improve patient safety continues to provide an example – both good and bad – to those who seek to implement system change. He may or may not have been the first person to attribute an infectious aetiology to childbed fever, but he was certainly not the last person to make a few unwise decisions regarding the best means of influencing healthcare worker behaviour. His story remains relevant and this anniversary offers an opportunity to reflect on current challenges in patient safety; what would Ignaz make of the current landscape in patient safety and hand hygiene? Here we compare and contrast Semmelweis’ innovations with current approaches to implementation of quality improvement programs, and other patient safety topics such as patient participation and public reporting.

## Disclosure of interest

None declared.

